# 1755. Hospitalizations for Respiratory Viruses among Children 5–17 Years of Age in the Pre-COVID-19 Pandemic Era, the New Vaccine Surveillance Network, 2016–2020

**DOI:** 10.1093/ofid/ofad500.1586

**Published:** 2023-11-27

**Authors:** Leila C Sahni, Justin Z Amarin, Tess Stopczynski, Andrew J Spieker, Laura S Stewart, Mary A Staat, Christina M Quigley, Rangaraj Selvarangan, Jennifer E Schuster, Marian G Michaels, John V Williams, Peter G Szilagyi, Geoffrey A Weinberg, Janet A Englund, Eileen J Klein, Benjamin R Clopper, Heidi L Moline, Vasanthi Avadhanula, Flor M Munoz, Pedro A Piedra, Natasha B Halasa, Julie A Boom

**Affiliations:** Baylor College of Medicine and Texas Children’s Hospital, Houston, Texas; Vanderbilt University Medical Center, Nashville, Tennessee; Vanderbilt University Medical Center, Nashville, Tennessee; Vanderbilt University Medical Center, Nashville, Tennessee; Vanderbilt University Medical Center, Nashville, Tennessee; Cincinnati Children’s Hospital Medical Center, Cincinnati, Ohio; Cincinnati Children's Hospital Medical Center, Cincinnati, Ohio; Children’s Mercy Kansas City, Kansas City, Missouri; Children’s Mercy Kansas City, Kansas City, Missouri; UPMC Children's Hospital of Pittsburgh, Pittsburgh, Pennsylvania; University of Pittsburgh, Pittsburgh, Pennsylvania; UCLA School of Medicine, Agoura Hills, California; University of Rochester School of Medicine & Dentistry, Rochester, NY; Seattle Children’s Hospital, Seattle, Washington; University of Washington School of Medicine, Seattle, Washington; US Centers for Disease Control & Prevention, Buffalo, New York; Centers for Disease Control and Prevention, Atlanta, Georgia; Baylor College of Medicine, Houston, Texas; Baylor College of Medicine, Houston, Texas; Baylor College of Medicine, Houston, Texas; Vanderbilt University Medical Center, Nashville, Tennessee; Texas Children’s Hospital, Houston, Texas

## Abstract

**Background:**

Respiratory viruses are a substantial cause of hospitalization in infants and young children; less is known about their contribution to ARI-associated hospitalizations in children ≥ 5 years of age.

**Methods:**

We conducted prospective, active surveillance among children ages 5–17 years hospitalized for acute respiratory illness (ARI) at 7 pediatric medical institutions comprising the New Vaccine Surveillance Network (NVSN) during December 1, 2016 through April 1, 2020. Children hospitalized for fever and/or respiratory symptoms were consented for enrollment. Demographic and clinical data were collected through parent/guardian interviews and chart abstraction. Mid-turbinate nasal and/or throat specimens were collected and tested using molecular methods for respiratory syncytial virus (RSV), influenza, parainfluenza viruses 1–4 (PIV), human metapneumovirus (HMPV), rhinovirus/enterovirus (RV/EV), adenovirus (AdV), and seasonal coronaviruses (CoV).

**Results:**

Of 6,992 eligible children, 4,011 (57%) were enrolled and 3,953 (99%) had respiratory specimens obtained. Median age of enrolled children was 8.9 years (IQR: 6.6, 12.2), 55% were male, 38% were non-Hispanic White, and 76% had ≥ 1 underlying medical condition (Table). Respiratory viruses were detected in 54% of patients hospitalized with ARI: RV/EV occurred most frequently (32%), followed by influenza (11%) and RSV (5%) (Figure). Respiratory virus coinfections were detected in 133 (3%) patients. Common symptoms reported included cough, congestion/runny nose, and fever, and a majority of children experienced dyspnea and wheezing. Median length of hospitalization was 2 days (IQR: 1,3); 687 (17%) patients were admitted to an intensive care unit, of whom 119 (17%) required intubation.

Figure. Detection and seasonality of respiratory viruses in hospitalized children 5–17 years, 2016–2020

**Figure:**
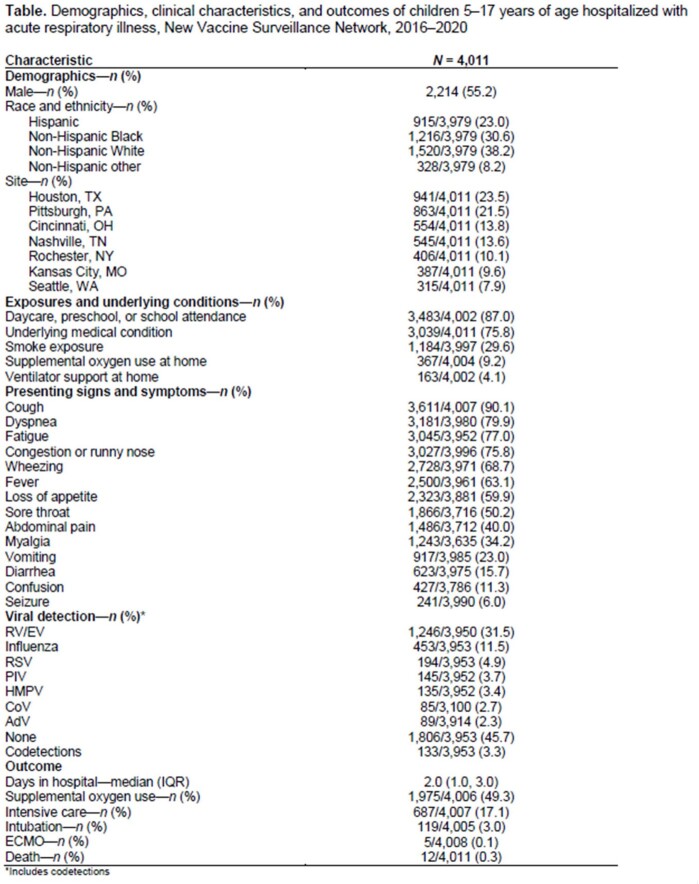

**Figure:**
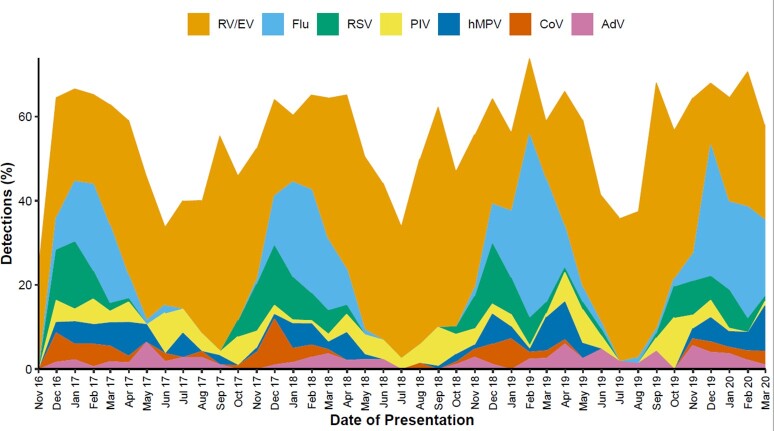

**Conclusion:**

Respiratory viruses were detected in more than half of ARI-related hospitalizations in children aged ≥ 5 years. This multi-center, multi-year, pre-pandemic assessment provides baseline data to evaluate for possible changing epidemiology of respiratory viruses after the onset of the COVID-19 pandemic.

**Disclosures:**

**Mary A. Staat, MD, MPH**, CDC: Grant/Research Support|Cepheid: Grant/Research Support|Merck: Grant/Research Support|NIH: Grant/Research Support|Pfizer: Grant/Research Support|Up-To-Date: Honoraria **Rangaraj Selvarangan, BVSc, PhD, D(ABMM), FIDSA, FAAM**, Abbott: Honoraria|Altona Diagnostics: Grant/Research Support|Baebies Inc: Advisor/Consultant|BioMerieux: Advisor/Consultant|BioMerieux: Grant/Research Support|Bio-Rad: Grant/Research Support|Cepheid: Grant/Research Support|GSK: Advisor/Consultant|Hologic: Grant/Research Support|Lab Simply: Advisor/Consultant|Luminex: Grant/Research Support **Marian G. Michaels, MD, MPH**, Merck: Grant/Research Support|Viracor: Grant/Research Support **John V. Williams, MD**, Merck: Grant/Research Support|Quidel: Board Member **Geoffrey A. Weinberg, MD**, Merck & Co: Honoraria **Janet A. Englund, MD**, Ark Biopharma: Advisor/Consultant|AstraZeneca: Advisor/Consultant|AstraZeneca: Grant/Research Support|GlaxoSmithKline: Grant/Research Support|Meissa Vaccines: Advisor/Consultant|Merck: Grant/Research Support|Moderna: Advisor/Consultant|Moderna: Grant/Research Support|Pfizer: Advisor/Consultant|Pfizer: Grant/Research Support|Sanofi Pasteur: Advisor/Consultant **Flor M. Munoz, MD, MSc**, CDC respiratory virus surveillance: Grant/Research Support|Gilead: Grant/Research Support|Moderna, sanofi, aztra zeneca, Merck, GSK: Advisor/Consultant|NIH: DSMB|NIH COVID-19 vaccines in pregnancy: Grant/Research Support|Pfizer Pediatric COVID-19 vaccines: Grant/Research Support|Pfizer, Dynavax, Monderna, Meissa, NIH: DSMB **Pedro A. Piedra, MD**, Ark Bioscience: Advisor/Consultant|Ark Bioscience: Grant/Research Support|GSK: Grant/Research Support|Icosavax: Advisor/Consultant|Icosavax: Grant/Research Support|Mapp Biologics: Grant/Research Support|Meissa Vaccines: Grant/Research Support|Moderna: Advisor/Consultant|Novavax: Advisor/Consultant|Novavax: Grant/Research Support|Sanofi-Pasteur: Grant/Research Support|Shionogi: Advisor/Consultant|Shionogi: Grant/Research Support|Takeda: Advisor/Consultant **Natasha B. Halasa, MD, MPH**, Merck: Grant/Research Support|Quidell: Grant/Research Support|Quidell: donation of kits|Sanofi: Grant/Research Support|Sanofi: vaccine support

